# Acute myeloid leukemia with t(4;12)(q12;p13): an aggressive disease with frequent involvement of *PDGFRA* and *ETV6*

**DOI:** 10.18632/oncotarget.23743

**Published:** 2017-12-15

**Authors:** Jingyi Li, Jie Xu, Lynne V. Abruzzo, Guilin Tang, Shaoying Li, M. James You, Gary Lu, Elias J. Jabbour, Qi Deng, Carlos E. Bueso-Ramos, L. Jeffrey Medeiros, C. Cameron Yin

**Affiliations:** ^1^ Department of Hematopathology, Tianjin First Center Hospital, Tianjin, China; ^2^ Department of Hematology, Tianjin First Center Hospital, Tianjin, China; ^3^ Current address: Department of Pathology, The Ohio State University, College of Medicine, Columbus, OH, USA; ^4^ Current address: Department of Biomedical Sciences, University of South Carolina School of Medicine, Greenville, SC, USA; ^5^ Department of Leukemia, The University of Texas MD Anderson Cancer Center, Houston, TX, USA

**Keywords:** acute myeloid leukemia, t(4;12)(q12;p13), PDGFRA, ETV6

## Abstract

We describe the clinical, morphologic, immunophenotypic and molecular genetic features of 15 cases of acute myeloid leukemia (AML) with t(4;12)(q12;p13). There were 9 men and 6 women, with a median age of 50 years (range, 17–76). Most patients had hypercellular bone marrow with a median blast count of 58% and multilineage dysplasia. Flow cytometry analysis showed myeloid lineage with blasts positive for CD13, CD33, CD34, CD38, CD117 and HLA-DR. Interestingly, aberrant CD7 expression was detected in 12/14 cases, and myeloperoxidase was either negative (3/15) or positive in only a small subset of the blasts (12/15). t(4;12)(q12;p13) was detected at time of initial diagnosis in 4 and at relapse or progression in 9 patients. The initial karyotype was unknown in 2 cases. FISH analysis showed *PDGFRA-ETV6* rearrangement in all 7 cases assessed. FLT3 ITD was detected in 2/11 cases and *IDH2* and *JAK2* mutation were each detected in 1/2 cases assessed. There were no mutations of *KRAS* (0/8), *NRAS* (0/8), *CEBPA* (0/3), *KIT* (0/3), *NPM1* (0/3) or IDH1 (0/2). All patients received aggressive multiagent chemotherapy; 7 patients additionally received stem cell transplantation. With a median follow-up of 10 months (range, 6–51), 13 patients died of AML, 1 patient had persistent disease, and 1 patient was lost to follow-up. In summary, AML with t(4;12)(q12;p13) is usually associated with myelodysplasia, aberrant CD7 expression, weak of absent myeloperoxidase expression, frequent *PDGFRA-ETV6* fusion, and an aggressive clinical course. The molecular findings suggest that there may be a role for tyrosine kinase inhibitors in patient management.

## INTRODUCTION

Conventional cytogenetic analysis plays a pivotal role in the risk stratification of acute myeloid leukemia (AML). The karyotype predicts response to induction therapy, risk of relapse, and overall survival. Acute myeloid leukemia with t(4;12) (q12;p13) has rarely been reported. Harada et al. described three cases of AML with t(4;12) (q12;p13) as the sole cytogenetic abnormality in 1995 [1]. He stated that the incidence of t(4;12)-positive AML was 0.6%. In 1999, Cools et al. identified the genes involved in this translocation: *ETV6* (ETS Translocation Variant 6, at 12p13) and *BTL* (Brx-like Translocated in Leukemia, later renamed *CHIC2*, at 4q12) [2]. Scattered case reports and small case series have been reported since then with a total of 25 cases reported to date [1–17]. Some reports have emphasized the suboptimal responses of patients treated using only standard chemotherapy regimens, with the chromosomal abnormality reappearing shortly after achievement of complete remission [4, 12, 13, 15]. The partner genes involved in this translocation were only explored in a few studies, and the molecular abnormalities were rarely explored. Here we report the clinical, morphologic, immunophenotypic and molecular genetic features of 15 AML cases with t(4;12) (q11;p13), the largest series to date.

## RESULTS

### Clinical findings

We identified 15 patients with AML associated with t(4;12) (q12;p13) seen at our institution from January 1, 1990 to December 31, 2016. The clinical and laboratory data are summarized in Table 1. There were 9 men and 6 women with a median age of 50 years (range, 17–76) at time of initial diagnosis. Upon presentation at our institution, laboratory evaluation showed anemia in 14 patients (median hemoglobin, 9.7 g/dL; range, 6.7-13.1 g/dL; reference range, 14.0-18.0 g/dL for men and 12.0-16.0 g/dL for women); thrombocytopenia in 11 patients and thrombocytosis in 1 patient (median platelet count, 42 × 10^3^/μL; range, 4-748×10^3^/μL; reference range, 140-440 × 10^3^/μL); and leukopenia in 7 and leukocytosis in 5 patients (median white blood cell count, 4.7 × 10^3^/μL; range, 1.2-87.3 × 10^3^/μL; reference range, 4.0-11.0 × 10^3^/μL). The serum lactate dehydrogenase level was elevated in 9 patients (median, 694 IU/L; range, 217-7139 IU/L; reference range, 313-618 IU/L), and the β2-microglobulin level was elevated in 10 of 11 patients assessed (median, 2.9 mg/L; range, 1.8-7.8 mg/L; reference range, 0.6-2.0 mg/L). Only 1 patient had B-symptoms (case 15). Three patients developed extramedullary involvement during their disease course (cases 1, 2 and 4). No patient had lymphadenopathy or hepatosplenomegaly.

**Table 1:** Clinical features, treatment, and outcome of AML patients with t(4;12)(q12;p13)

# Sex/Age	Diagnosis (WHO)	Prior history	WBC (10^3^/μL)	Hb (g/dL)	Platelet (10^3^/μL)	LDH (IU/L)	β2M (mg/L)	Chemotherapy	BMT	Outcome (FU^*^)
1 M/65	AML-MRC	MDS	2.9	10.3	99	332	7.3	I, C. Fl	Yes	DOD (8)
2 F/36	AML with minimal differentiation	–	45.0	8.0	16	7139	NA	I, C	Yes	DOD (7)
3 M/35	Acute erythroid leukemia	–	1.4	10.4	4	217	NA	I, C	Yes	DOD (16)
4 F/45	AML-MRC	PV	9.8	13.1	201	580	2.9	I, C, Mi, E, Fl, G-CSF	Yes	DOD (51)
5 M/48	AML without maturation	–	87.3	10.4	57	1032	4.1	I, C, Fl, Cy, Top, ABT-751	No	DOD (9)
6 F/58	AML with minimal differentiation	–	3.1	10.7	338	495	1.8	I, C	Yes	DOD (51)
7 M/57	AML-MRC	MDS	8.6	8.6	39	2679	2.5	Dec, C, Clo	No	DOD (8)
8 F/76	AML without maturation	–	2.6	6.7	95	692	5.3	I, C, PKC-412	No	DOD (6)
9 M/67	AML-MRC	CMML	3.9	8.4	29	875	2.5	E, R, I, C, My, RAD-001	No	DOD (7)
10 F/54	Acute myelomonocytic leukemia	–	1.2	9.2	17	694	NA	I, C, My, Fl, Das, AZD-1152	No	DOD (12)
11 M/17	AML-MRC	–	3.7	9.2	38	461	NA	C, Dau, E	No	Lost FU
12 M/41	AML without maturation	–	19.5	12.6	171	3025	4.0	Dau, I, C, My, Mi, E, Top, OSI-211	No	DOD (19)
13 F/50	AML-unknown	–	11.5	9.1	17	1779	7.8	I, C, Fl, My, E, Cy, Dec	No	DOD (11)
14 M/24 15 M/56	AML without maturation AML-MRC	– PV	23.3 4.7	9.7 9.7	42 748	595 1093	2.9 2.8	Clo, I, C I, C, HU, Ana, Rux	Yes Yes	DOD (6) PV (40)

^*^Follow-up (months) from time of initial diagnosis

Abbreviations: ABT-751, microtubule inhibitor; AML, acute myeloid leukemia; Ana, anagrelide; AZD-1152, aurora kinase inhibitor; β2M, β2-microglobulin; BMT, bone marrow transplantation; C, cytarabine; Clo, clofarabine; CMML, chronic myelomonocytic leukemia; Cy, cyclophosphamide; Das, dasatinib; Dau, daunorubicin; Dec, decitabine; DOD, died of disease; E, etoposide; F, female; FAB, French-American-British; Fl, fludarabine; FU, follow-up; Hb, hemoglobin; HU, hydroxyurea; I, idarubicin; LDH, lactate dehydrogenase; M, male; MDS, myelodysplastic syndrome; Mi, mitoxantrone; MRC, myelodysplasia-related changes; My, mylotarg; NA, not available; OSI-211, liposomal topoisomerase inhibitor; PKC-412, midostaurin; PV, Polycythemia vera; R, rituxan; RAD-001, mTOR inhibitor; Rux, Ruxolitinib; Top, topotecan; WBC, white blood cell; WHO, World Health Organization

### Morphologic findings

The World Health Organization diagnostic category for the study group included: 6 cases of AML with myelodysplasia-related changes that evolved from polycythemia vera (*n* = 2), myelodysplastic syndrome (*n* = 2), chronic myelomonocytic leukemia (*n* = 1), and 1 de novo; 4 cases of AML without maturation (French-American-British [FAB] classification M1); 2 cases of AML with minimal differentiation (FAB M0), and 1 case each of acute myelomonocytic leukemia (FAB M4) and acute erythroid leukemia (FAB M6). The classification of 1 case is unknown and slides were not available for review.

**Figure 1:**
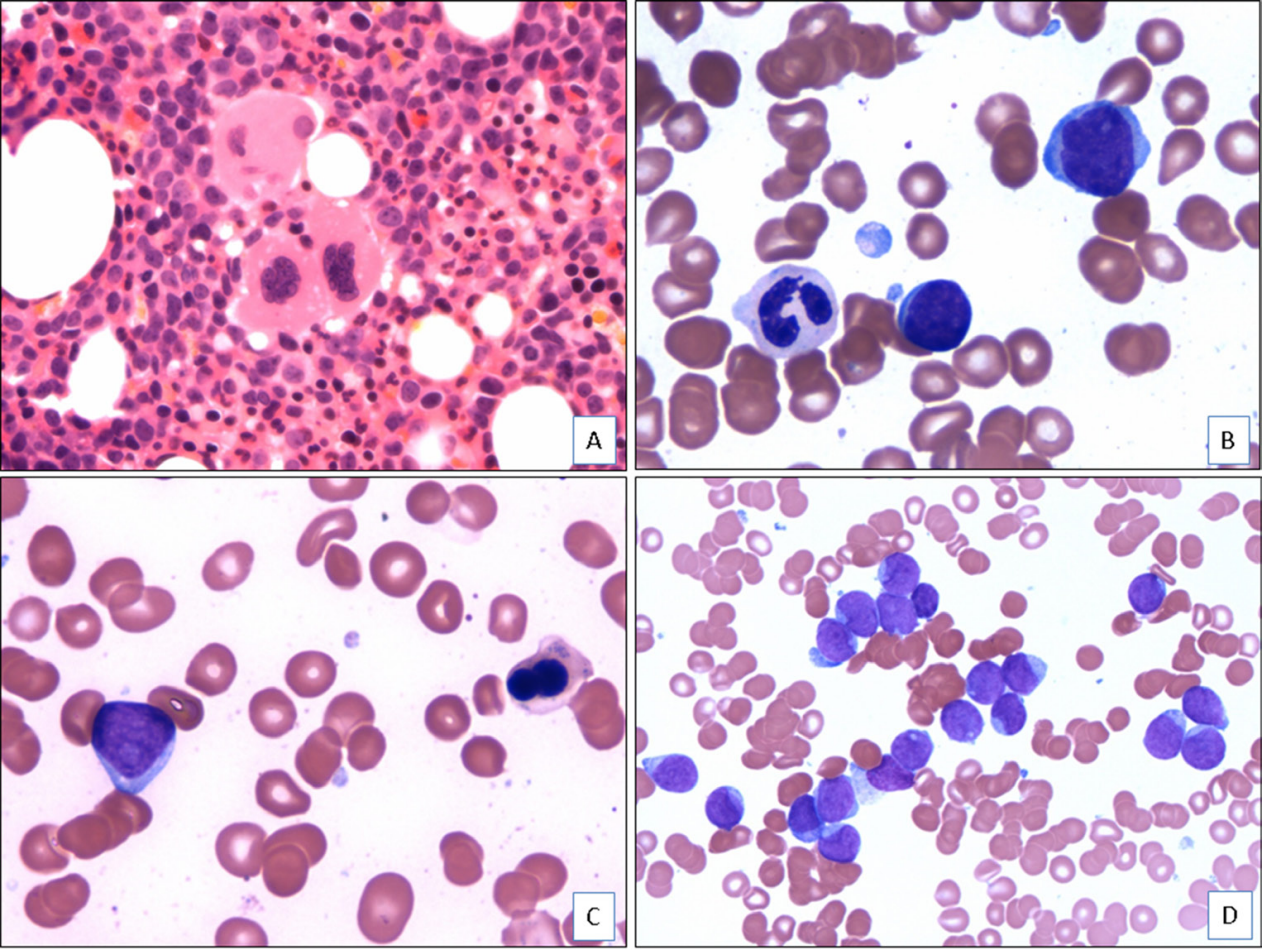
Morphologic features of AML with t(4;12)(q12;p13) (case 5) (**A**). The bone marrow core biopsy shows a hypercellular bone marrow with dysplastic megakaryocytes and increased immature cells (H&E, x400). (**B**). The aspirate smear shows dysgranulopoiesis and increased blasts (Wright-Giemsa, x1000). (**C**). The aspirate smear shows dyserythropoiesis and increased blasts (Wright-Giemsa, x1000). (**D**). The aspirate smear shows numerous blasts that are small to intermediate in size with fine chromatin, occasional prominent nucleoli, and scant basophilic cytoplasm (Wright-Giemsa, x500).

The bone marrow was generally hypercellular (median cellularity, 75%), with a median blast count of 58% (range, 34%–91%). In most cases, the blasts were small to intermediate in size with fine chromatin, occasional small nucleoli, and scant basophilic cytoplasm. Dysplasia was observed in 10 cases; the other 5 cases had too few maturing cells to assess dysplasia (Figure 1). Only 1 case, an AML arising from polycythemia vera, showed eosinophilia (case 4). None showed basophilia.

### Immunophenotypic findings

Immunophenotypic analysis by flow cytometry demonstrated the presence of a distinct myeloid blast population in all cases. The blasts expressed CD13 (14/15, 93%), CD33 (14/15, 93%), CD34 (14/14, 100%), CD38 (14/14, 100%), CD117 (14/14, 100%) and HLA-DR (13/14, 93%). The blasts in small subsets of cases were also positive for CD64 (6/14, 43%), CD15 (3/14, 21%), CD14 (1/14, 7%) and CD56 (1/14, 7%). All cases were negative for CD3 (surface or cytoplasmic), CD5, CD10, CD19, CD20, and terminal deoxynucleotidyl transferase (TdT). Myeloperoxidase was either negative (*n* = 3) or positive in only a small subset of blasts (*n* = 12). Aberrant CD7 expression was detected in 12/14 (86%) cases. Interestingly, acquisition of the t(4;12) was associated with up-regulation of CD7 expression (cases 5, 6 and 7) and down-regulation of myeloperoxidase expression (cases 3, 5 and 7) in patients who acquired the t(4;12) during relapse or disease progression.

### Cytogenetic findings

The results of conventional cytogenetic and FISH analyses are summarized in Table 2. The t(4;12) (q12;p13) was identified at time of initial diagnosis in 4 patients (cases 4, 8, 11 and 14), at time of progression from MDS to AML in 3 patients (cases 1, 7 and 9) , and at time of relapse in 6 patients (cases 3, 5, 6, 10, 12 and 15). The initial karyotypes were unknown in two cases (cases 2 and13).

**Table 2:** Cytogenetic and molecular findings of AML with t(4;12)(q12;p13)

Case	Karyotype	FISH	Gene Mutations
1	46,XY,t(4;12)(q12;p13)[7]/46,XY,t(4;12) (q12;p13),del(7)(q21q32)[6] /53,XY,+6,+8,+10,+11,+12,+14,+19[6]	*PDGFRA-ETV6*	*KRAS-, NRAS-*
2	46,XX,t(4;12) (q12;p13),del(9) (q22)[20]	NA	NA
3	41-42,XY,add(2)(q44),-3,i(3)(q10),t(4;12)(q12;p13),del(5)(p14),del(5) (q13), -7,-9,del(11)(q12),add(14) (p11),-15,-16,-17,-18,-20-,21,-22,+6mar [cp20]	NA	NA
4^*^	46,XX,t(4;12)(q12;p13)[1]/46,XX [29] 46,XX,t(2;13)(p21;q14),t(4;12)(q12;p13)[17]/46,sl,del(9)(q13q22)[3]	*PDGFRA-ETV6*	*CEBPA-, FLT3-, KIT-, KRAS-, NPM1-, NRAS-*
5	46,XY,t(4;12)(q12;p13)[17]/46,XY [3]	*PDGFRA-ETV6*	*FLT3-ITD+*
6	46,XX,t(4;12)(q12;p13)[1]/46,XX,sl,del(16)(q22)[11] /46,sl,t(1;21)(q11;q11.1)[4]/46,XX [3]	*PDGFRA-ETV6*	*FLT3-, KRAS-, NRAS-*
7	45,XY,t(4;12)(q12;p13),der(13;15)(q10;q10),del(20)(q11.2q13.3)[20]	*PDGFRA-ETV6*	*FLT3-, JAK2-, KRAS-, NRAS-*
8	46,XX,t(4;12)(q12;p13)[19]	NA	*FLT3-*
9	46,XY,t(4;12)(q12;p13),-7,+mar [13]/46,XY [7]	NA	*FLT3-, KRAS-, NRAS-*
10^*^	46,XX,t(4;12)(q12;p13)[11]/46,XX [7] 46,XX,t(4;12)(q12;p13),t(9;11)(p22;q23),del(10)(q22q24)[12]	*PDGFRA-ETV6*	*FLT3-, KRAS-, NRAS-*
11	46,XY,t(4;12)(q12;p13)[20]	*PDGFRA-ETV6*	*FLT3-*
12	46,XY,t(4;12)(q12;p13)[12]/46,XY,t(1;4)(q25;p14),del(10)(q25),-20,+mar[3]/ 46,XY,inv(4)(p16q12)[3]/46,XY [2]	NA	NA
13	46,XX,t(4;12)(q12;p13),t(10;13)(q22;q12)[12]/46,sl,del(2)(q33q37)[3]/ 46,XX,t(4;12)(q12;p13),t(10;13)(q22;q12)[cp5]	NA	*FLT3-ITD+*
14 15	46,XY,t(4;12)(q12;p13),t(9;14)(p13;q13)[19]/46,XY [1] 46,XY,t(4;12)(q12;p13)[20]	NA NA	*CEBPA-, FLT3-, IDH1-, IDH2-, KIT-, KRAS-,* *NPM1-, NRAS-* *IDH2+, JAK2+, CEBPA-, FLT3-, IDH1-, KIT-, KRAS-, NPM1-, NRAS-*

^*^These two patients had t(4;12) as a sole abnormality at initial diagnosis and acquired additional cytogenetic aberrations at relapse (case 4) or progression (case 10).

Abbreviations: AML, acute myeloid leukemia; NA, not available

**Figure 2:**
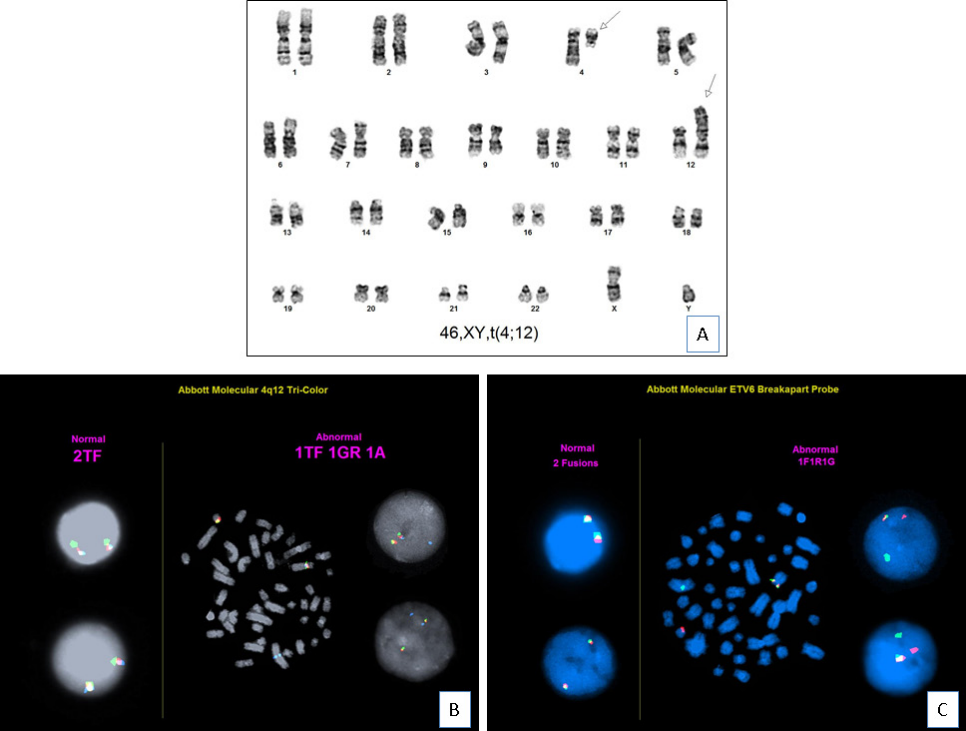
Cytogenetic findings (case 11) (**A**). Chromosomal analysis shows a karyotype of 46,XY,t(4;12)(q12;p13)[20]. (**B**). FISH analysis using *PDGFRA* tri-color break-apart probe (*FIP1L1* in green, *CHIC2* in red and *PDGFRA* in aqua) shows 1 triple fusion, 1 green-red fusion, and 1 separate aqua signal, which confirms *PDGFRA* gene rearrangement (translocation of aqua signal to derivative chromosome 12). (**C**). FISH analysis using *ETV6* dual-color break-apart probe shows *ETV6* gene rearrangement (translocation of red signal to derivative chromosome 4).

The t(4;12)(q12;p13) was present as the sole cytogenetic abnormality in 7 patients (cases 4, 5, 8, 10, 11, 12 and 15) (Figure 2), as one of two abnormalities (simple abnormal karyotype) in 3 patients (cases 1, 2 and 14), and as a part of complex karyotype (≥ 3) in 5 patients (cases 3, 6, 7, 9 and 13). Two patients in whom t(4;12) was a sole abnormality at initial diagnosis subsequently acquired additional cytogenetic aberrations at time of relapse (case 4) or progression (case 10).

FISH analyses were performed using break-apart probes for *PDGFRA* and *ETV6* on cultured bone marrow cells, respectively. For *PDGFRA*, tri-color break-apart probe (*FIP1L1* in green, *CHIC2* in red and *PDGFRA* in aqua) showed 1 triple fusion, 1 green-red fusion, and 1 separate aqua signal, which confirmed *PDGFRA* gene rearrangement (translocation of aqua signal to derivative chromosome 12). For *ETV6*, translocation of red signal to derivative chromosome 4 confirmed *ETV6* rearrangement. In summary, all 7 cases assessed (cases 1, 4, 5, 6, 7, 10 and 11) showed both *PDGFRA* and *ETV6* rearrangements (Figure 2).

### Molecular findings

Molecules studies revealed a *FLT3* internal tandem duplication (ITD) in 2 of 11 cases assessed (cases 5 and 13). *IDH2* and *JAK2* mutation was each detected in 1 of 2 cases assessed (case 15). No cases assessed showed mutations of *KRAS* (*n* = 8), *NRAS* (*n* = 8), *CEBPA* (*n* = 3), *KIT* (*n* = 3), *NPM1* (*n* = 3), or *IDH1* (*n* = 2) (Table 2).

### Clinical outcome

All patients received multiagent chemotherapy; 5 patients also were treated with investigational drugs including ABT-751 (microtubule inhibitor), AZD-1152 (aurora kinase inhibitor), OSI-211 (liposomal topoisomerase inhibitor), PKC-412 (midostaurin, tyrosine kinase inhibitor), and RAD-001 (mTOR inhibitor). Seven patients additionally underwent hematopoietic stem cell transplantation. Two patients received tyrosine kinase inhibitor therapy: patient 10 received dasatinib and patient 15 received ruxolitinib. Of the 14 patients with clinical follow-up data, 4 patients were refractory to therapy and 10 patients relapsed shortly after first complete remission with a median time to first relapse of 4 months. With a median follow-up of 10 months from time of initial diagnosis (range, 6–51) or 6 months from the occurrence of t(4;12) (range, 2–51), 13 patients died of AML, 1 patient (case 15) had persistent disease, and 1 patient was lost to follow-up. Among the 14 patients with follow-up data available, 7 (50%) patients died within 1 year, 10 (71%) patients died within 2 years, and 13 (93%) patients died within 5 years.

## DISCUSSION

The t(4;12)(q12;p13) is a rare recurrent cytogenetic abnormality in AML, reported previously in 25 cases as case reports or small series, with limited molecular genetic analysis [1–17]. We present the clinical, morphologic, immunophenotypic and molecular genetic features of 15 AML cases with t(4;12)(q12;p13) evaluated at a single institution, the largest series to date.

In the initial study of 3 cases of AML with t(4;12), Harada and colleagues described the blasts as having “pseudo-lymphoid” morphology and a background of trilineage dysplasia, basophilia, and eosinophilia were common. The results in this series, in part, confirm these findings. The blasts were small to intermediate in size and myeloperoxidase expression was present in only a subset of blasts. These blasts do resemble, in part, lymphoblasts. We also observed mutlilineage dysplasia in all cases in which the number of maturing hematopoietic cells was adequate to evaluate. In addition, some patients had a history of a myelodysplastic syndrome or myeloproliferative neoplasm. However, no cases in this study were associated with basophilia and only one patient with a history of polycythemia vera had eosinophilia.

Recurrent chromosomal rearrangements that generate oncogenic fusion genes or deregulate the expression of proto-oncogenes and/or tumor suppressor genes play an essential role in the development and progression of hematologic neoplasms. In our study, the t(4;12) was found as both a primary abnormality at diagnosis and a secondary abnormality associated with relapse or progression. In an earlier study, Cools et al. identified the genes involved in this translocation to be *ETV6* (at 12p13) and *BTL* (at 4q12) [2]. The partner genes involved in this translocation were rarely explored since then. In all 7 of our cases assessed by FISH, we identified rearrangements of *PDGFRA* and *ETV6*, suggesting a role in the pathogenesis of AML with t(4;12). Located as 4q12, *PDGFRA* is a member of the class III receptor tyrosine kinase family [18, 19]. It contains an extracellular immunoglobulin-like domain, a transmembrane domain with an inhibitory juxtamembrane WW-like domain, and an intracellular kinase domain [20]. PDGFRA activates intracellular tyrosine kinase signaling pathway by forming homodimer or heterodimer with PDGFRB [21]. Hematologic malignancies associated with *PDGFRA* rearrangement commonly manifest as myeloid or lymphoid neoplasms with eosinophilia [19]. Its most common partner gene is *FIP1L1.* An approximately 800 kb interstitial chromosomal deletion juxtaposes *FIP1L*1 and *PDGFRA* resulting in a gain-of-function fusion protein with signal-independent kinase activity and increases cell proliferation and survival [9].

The *ETV6* gene (previously called *TEL*), located at 12p13, is a member of the ETS family of transcription factors. It contains two important domains: the HLH (helix-loop-helix) domain, which mediates protein-protein interactions, and the ETS DNA binding domain [22]. *ETV6* is the major target of translocations involving 12p13 in hematopoietic malignancies, and is frequently rearranged in both myeloid and lymphoid neoplasms [22]. Translocations involving *ETV6* generate oncogenic fusion proteins or ectopic promoters [1, 2, 6]. One study has suggested that the transcriptional activation of *PDGFRA* by the positive effect of *ETV6* translocation may be involved in leukemogenesis of these cases [15].

In keeping with earlier studies, acquisition of the t(4;12) appears to be associated with up-regulation of CD7 and down-regulation of myeloperoxidase expression. Interestingly, in patients with sequential bone marrow specimens who acquired the t(4;12) upon relapse or disease progression, increased CD7 and decreased myeloperoxidase expression coincided with acquisition of the translocation. We speculate that the presence of the fusion protein, via downstream pathways, down-regulate myeloperoxidase and up-regulate CD7 although we have no data to explain the possible mechanisms. Moreover, the minimal expression or complete absence of myeloperoxidase and aberrant CD7 expression, along with the morphology of the blasts, can present a challenging differential diagnosis with mixed phenotype acute leukemia (myeloid/T). However, none of the cases showed conclusive evidence of T-cell lineage manifested by lack of surface or cytoplasmic CD3 expression. Therefore, none of these cases met the diagnostic criteria for precursor T immunophenotype.

Some of the earlier studies of AML with t(4;12) have reported the difficulties of using only standard chemotherapy regimens in treating these patients [4, 12, 13, 15]. Others have suggested that these patients may respond to intensive chemotherapy regimens or hematopoietic stem cell transplantation [2, 13, 16]. Our data also suggest that the t(4;12), either as a sole abnormality or as part of a complex karyotype, portends a poor outcome. In the patient cohort presented, despite treatment with multiagent chemotherapy, new investigational agents, and/or hematopoietic stem cell transplantation, most patients failed to achieve a complete remission or only had a brief remission of short duration with multiple relapses and a poor outcome.

In summary, we have described 15 AML cases associated with t(4;12)(q12;p13), the largest series to date. Our results show that AML with t(4;12) (q12;p13) is a distinct entity with an aggressive clinical course and characteristic, although not specific, morphologic and immunophenotypic findings. The t(4;12)(q12;p13) can occur at initial diagnosis or at relapse or disease progression, and frequently involves *PDGFRA* and *ETV6*. There may be a role for tyrosine kinase inhibitor therapy in patients with this disease.

## MATERIALS AND METHODS

### Case selection

We searched the database of the Department of Hematopathology at The University of Texas MD Anderson Cancer Center from January 1, 1990 to December 31, 2016 and identified 15 patients with AML associated with t(4;12)(q11;p13). The diagnosis of AML was based on morphologic and immunophenotypic criteria as specified in the revised World Health Organization classification [23]. Clinical and laboratory data were obtained by review of the medical records. The study was conducted under an Internal Review Board-approved protocol.

### Morphologic examination

We reviewed Wright Giemsa-stained peripheral blood smears, bone marrow aspirate smears and touch imprints, as well as H&E-stained core biopsy and clot sections in all cases. A manual 100-cell or 500-cell differential count was performed on peripheral blood smear and bone marrow aspirate smear, respectively. Cytochemical stains for myeloperoxidase were performed on aspirate smears in most cases using conventional methods.

### Immunophenotypic analysis

Immunophenotypic analysis using multicolor flow cytometry was performed on bone marrow aspirates as previously described [24]. The panel of monoclonal antibodies used included reagents specific for CD3 (surface and cytoplasmic), CD5, CD7, CD10, CD13, CD14, CD15, CD19, CD20, CD33, CD34, CD38, CD45, CD56, CD64, CD117, myeloperoxidase, HLA-DR, and terminal deoxynucleotidyl transferase. All antibodies were purchased from Becton Dickinson Biosciences (San Jose, CA). Analysis was performed using a FACScan or FACSCalibur cytometer (Becton Dickinson Biosciences). An isotype-matched control was used for each antibody.

### Conventional cytogenetic and FISH analyses

In all cases, conventional cytogenetic analysis was performed on metaphase cells prepared from bone marrow aspirates cultured for 24 or 48 hours without mitogens, using standard techniques. Giemsa-banded metaphases were analyzed, and the results were reported using the International System for Human Cytogenetic Nomenclature, 2016 (ISCN, 2016)

Fluorescence *in situ* hybridization (FISH) analysis was performed on interphase nuclei obtained from cultures of bone marrow aspirates using probes for *PDGFRA* (tri-color break-apart probe, Abbott Molecular/Vysis, Des Plaines, IL) and *ETV6* (dual-color break-apart probe, Abbott Molecular/Vysis), respectively, using standard techniques. The positive cut-off values established in our laboratory are 4.7% for *PDGFRA* rearrangement and 3.2% for *ETV6* rearrangement.

### Molecular studies

Genomic DNA extracted from bone marrow aspirates was PCR amplified and subject to mutational analysis for *CEBPA*, *IDH1* (exon 4), *IDH2* (exon 4), *KIT* (exon 17), *NPM1* (exon 12), and *TP53* (exons 2–11) by direct Sanger sequencing on an ABI Prism 3100 Genetic Analyzer (Applied Biosystems, Foster City, CA), or *JAK2* V617F and codons 12, 13 and 61 of *KRAS* and *NRAS* by pyrosequencing using a PSQ HS 96 Pysosequencer (Biotage, Uppsala, Sweden), as described previously [25].

A fluorescence-based multiplex PCR was used to detect ITD and D835 point mutation of the *FLT3* gene using genomic DNA. For D835, the PCR products were digested with EcoRV restriction enzyme that cuts only the wild type sequence. The PCR products were then subjected to capillary electrophoresis on an ABI Prism 3100 Genetic analyzer to distinguish wild and mutant genotypes [25].

## References

[1] Harada H, Asou H, Kyo T, Asaoku H, Iwato K, Dohy H, Oda K, Harada Y, Kita K, Kamada N (1995). A specific chromosome abnormality of t(4;12) (q11-12;p13) in CD7+ acute leukemia. Br J Haematol.

[2] Cools J, Bilhou-Nabera C, Wlodarska I, Cabrol C, Talmant P, Bernard P, Hagemeijer A, Marynen P (1999). Fusion of a novel gene, BTL, to ETV6 in acute myeloid leukemias with a t(4;12) (q11-q12;p13). Blood.

[3] Den Nijs van Weert JI, Beverstock GC, Kievits T, Haak HL, Havik-Boggard FC, Leeksma CH (1989). Der(1) (t(1;9): A specific chromosome abnormality in polycythemia vera? Cytogenetic and *in situ* hybridization studies. Cancer Genet Cytogenet.

[4] Harada H, Harada Y, Eguchi M, Dohy H, Kamada N (1997). Characterization of acute leukemia with t(4;12). Leuk Lymphoma.

[5] Sainty D, Arnoulet C, Mozziconacci MJ, De Pina JJ, Garnotel E, Lafage-Pochitaloff M (1997). t(4;12) (q11;p13) in a CD7-negative acute myeloid leukaemia. Br J Haematol.

[6] Ma SK, Lei AK, Au WY, Wan TS, Chan LC (1997). CD7+ acute myeloid leukemia with ‘mature lymphoid’ blast morphology, marrow basophilia and t(4;12) (q12;p13). Br J Haematol.

[7] Hamaguchi H, Nagata K, Yamamoto K, Kobayashi M, Takashima T, Taniwaki M (1999). A new translocation, t(2;4;12) (p21;q12;p13), in CD7-positive acute myeloid leukemia: a variant form of t(4;12). Cancer Genet Cytogenet.

[8] Nathan PC, Chun K, Abdelhaleem M, Malkin D (2001). Isochromosome (17) (q10) and translocation t(4;12) (q12;p13) in a child with acute myeloid leukemia. Cancer Genet Cytogenet.

[9] Cools J, Mentens N, Odero MD, Peeters P, Wlodarska I, Delforge M, Hagemeijer A, Marynen P (2002). Evidence for position effects as a variant ETV6-mediated leukemogenic mechanism in myeloid leukemias with t(4;12) (q11-q12;p13) or t(5;12) (q31;p13). Blood.

[10] Chauffaille Mde L, Fermino FA, Pelloso LA, Silva MR, Bordin JO, Yamamoto M (2003). t(4;12) (q11;p13): a rare chromosomal translocation in acute myeloid leukemia. Leuk Res.

[11] Kuchenbauer F, Schoch C, Holler E, Haferlach T, Hiddemann W, Schnittger S (2005). A rare case of acute myeloid leukemia with a CHIC2-ETV6 fusiongene and multiple other molecular aberrations. Leukemia.

[12] Al-Kali A, Cherry M, Kimmell K, Holter J, Kern W, Gehrs B, Ozer H, Selby G (2010). A case of acute myeloid leukemia initially treated as chronic lymphocytic leukemia: what do we know about t(4;12) (q12;p13)?. Cancer Genet Cytogenet.

[13] Manabe M, Nakamura K, Inaba A, Fujitani Y, Kosaka S, Yamamura R, Inoue A, Hino M, Senzaki H, Ohta K (2010). A rare t(4;12)(q12;p13) in an adolescent patient with acute myeloid leukemia. Cancer Genet Cytogenet.

[14] Di Giacomo D, La Starza R, Barba G, Pierini V, Baldazzi C, Storlazzi CT, Daniele G, Forghieri F, Borlenghi E, Testoni N, Mecucci C (2015). 4q12 translocations with GSX2 expression identify a CD7(+) acute myeloid leukaemia subset. Br J Haematol.

[15] Abe A, Mizuta S, Okamoto A, Yamamoto Y, Kameyama T, Mayeda A, Emi N (2016). Transcriptional activation of platelet-derived growth factor receptor a and GS homeobox 2 resulting from E26 transformation-specific variant 6 translocation in a case of acute myeloid leukemia with t(4;12) (q12;p13). Int J Lab Hematol.

[16] Kim KH, Kim MJ, Ahn JY, Park PW, Seo YH, Jeong JH (2016). Acute myeloid leukemia with t(4;12) (q12;p13): report of 2 cases. Blood Res.

[17] Patel UV, Arun SR, Mishra DK, Parihar M (2016). Acute myeloid leukemia with t(4;12) (q12;p13): A morphological dilemma. Indian J Pathol Microbiol.

[18] Reilly JT (2002). Class III receptor tyrosine kinases: role in leukaemogenesis. Br J Haematol.

[19] Bain BJ (2010). Myeloid and lymphoid neoplasms with eosinophilia and abnormalities of PDGFRA, PDGFRB or FGFR1. Haematologica.

[20] Irusta PM, Luo Y, Bakht O, Lai CC, Smith SO, DiMaio D (2002). Definition of an inhibitory juxtamembrane WW-like domain in the platelet-derived growth factor beta receptor. J Bio Chem.

[21] Kawagishi J, Kumabe T, Yoshimoto T, Yamamoto T (1995). Structure, organization, and transcription units of the human alpha-platelet-derived growth factor receptor gene, PDGFRA. Genomics.

[22] Bohlander SK (2005). ETV6: a versatile player in leukemogenesis. Semin Cancer Biol.

[23] Arber DA, Orazi A, Hasserjian R, Thiele J, Borowitz MJ, Le Beau MM, Bloomfield CD, Cazzola M, Vardiman JW (2016). The 2016 revision to the World Health Organization classification of myeloid neoplasms and acute leukemia. Blood.

[24] Yin CC, Medeiros LJ, Glassman AB, Lin P (2004). t(8;21) (q22;q22) in blast phase of chronic myelogenous leukemia. Am J Clin Pathol.

[25] Kanagal-Shamanna R, Bueso-Ramos CE, Barkoh B, Lu G, Wang S, Garcia-Manero G, Vadhan-Raj S, Hoehn D, Medeiros LJ, Yin CC (2012). Myeloid neoplasms with isolated isochromosome 17q represent a clinicopathologic entity associated with myelodysplastic/myeloproliferative features, a high risk of leukemic transformation, and wild-type TP53. Cancer.

