# Correction: Acute myeloid leukemia with t(4;12)(q12;p13): an aggressive disease with frequent involvement of PDGFRA and ETV6

**DOI:** 10.18632/oncotarget.26205

**Published:** 2018-09-28

**Authors:** Jingyi Li, Jie Xu, Lynne V. Abruzzo, Guilin Tang, Shaoying Li, M. James You, Gary Lu, Elias J. Jabbour, Qi Deng, Carlos E. Bueso-Ramos, L. Jeffrey Medeiros, C. Cameron Yin

**Affiliations:** ^1^ Department of Hematopathology, The University of Texas MD Anderson Cancer Center, Houston, TX, USA; ^2^ Department of Hematology, Tianjin First Center Hospital, Tianjin, China; ^3^ Current address: Department of Pathology, The Ohio State University, College of Medicine, Columbus, OH, USA; ^4^ Current address: Department of Biomedical Sciences, University of South Carolina School of Medicine, Greenville, SC, USA; ^5^ Department of Leukemia, The University of Texas MD Anderson Cancer Center, Houston, TX, USA

**This article has been corrected:** The correct author affiliation is given below:

^1^ Department of Hematopathology, The University of Texas MD Anderson Cancer Center, Houston, TX, USA

Original article: Oncotarget. 2018; 9:10987-10994. https://doi.org/10.18632/oncotarget.23743

